# Mentalization, Oxytocin, and Cortisol in the General Population

**DOI:** 10.3390/life13061329

**Published:** 2023-06-06

**Authors:** Edina Török, Oguz Kelemen, Szabolcs Kéri

**Affiliations:** 1 Department of Cognitive Science, Budapest University of Technology and Economics, 1111 Budapest, Hungary; etorok@edu.bme.hu; 2 Department of Behavioral Sciences, Albert Szent-Györgyi Medical School, University of Szeged, 6722 Szeged, Hungary; kelemen.oguz@med.u-szeged.hu; 3 National Institute of Mental Health, Neurology, and Neurosurgery, 1145 Budapest, Hungary; 4 Department of Physiology, Albert Szent-Györgyi Medical School, University of Szeged, 6720 Szeged, Hungary

**Keywords:** mentalization, oxytocin, cortisol, biological motion, emotion regulation

## Abstract

Although evidence suggests the role of oxytocin and cortisol in social cognition and emotion regulation, it is less known how their peripheral levels are related to social perception (biological motion detection) and mentalization (self-reflection, emotional awareness, and affect regulation) in the general population. We assessed 150 healthy individuals from the general community on a mentalization questionnaire, a scale measuring the intensity of positive and negative emotions, and measured oxytocin and cortisol levels in the saliva. Oxytocin but not cortisol level and biological motion detection predicted mentalization abilities. There was a positive correlation between mentalization and positive emotions and between mentalization and biological motion detection. These results suggest that oxytocin, but not cortisol, plays a role in low-level perceptual and self-reflective aspects of social cognition.

## 1. Introduction

During mentalization (Theory of Mind, ToM), individuals use mental state terms referring to cognitive and affective processes (intentions, beliefs, feelings, and motivations) to interpret their own and others’ behavior and experiences [1,2,3]. Mentalization can be spontaneous, fast, or consciously controlled, driven by internal or external features of ourselves and others [4]. In addition to the plain understanding of intentional stances (ToM), mentalization is crucially implicated in higher-level self-reflection, discrimination of internal states and external reality, emotional awareness, and affective regulation. For the development of appropriate mentalization, a stable attachment to significant others is essential, linking this concept to general aspects of social cognition and affiliation [5,6,7].

The biological basis of mentalization is multidimensional, including a widespread medial prefrontal and temporoparietal brain network and sophisticated neurohormonal regulation [3,8,9]. A critical factor is oxytocin, a peptide hormone produced in the hypothalamus and several other brain parts as a neurotransmitter, essential in childbirth, lactation, and maternal behavior. Furthermore, its importance has been demonstrated in stress coping, fear learning, creating pair bonds, attachment, social perception (e.g., faces expressing emotions), trust, and attributing mental states to others during mentalization in normal and clinical conditions [10,11,12]. In addition to human research, there is an increasing interest in the role of oxytocin in social bonding and welfare in domesticated animals, with a particular reference to dogs, but the results are heterogeneous and non-conclusive [13,14,15]. 

The relationship between oxytocin, mentalization, and social cognition in a broader sense is controversial and complex [16,17]. We first discuss the link between social cognition and baseline peripheral oxytocin levels in the blood plasma and saliva. In patients with schizophrenia, lower oxytocin concentrations are associated with decreased trust, impairments in identifying facial emotions, and deficits in the affective component of mentalization [18]. Patients with borderline personality disorder display mentalization problems and exhibit changes in plasma oxytocin levels, especially concerning childhood trauma and activity [19,20]. Specifically, plasma oxytocin correlated negatively with experiences of childhood emotional neglect and abuse [20]. Meanwhile, a positive relationship was revealed between plasma oxytocin levels and the activity measure of the Zuckerman–Kuhlman Personality Questionnaire (ability to relax when opportunity arises, preference for challenging tasks, and high energy level), which is associated with better social adaptation [19]. Patients with borderline personality disorder also displayed low reactivity of saliva oxytocin during stress (public speaking and social evaluation): the stress situation did not induce increased oxytocin secretion in the patient group, and lower oxytocin levels correlated with anxiety and anger [21].

A pertinent question is whether peripheral oxytocin is a reliable biological marker. According to previous studies, saliva and plasma oxytocin levels did not correlate in men [22,23], whereas, in breast- and formula-feeding mothers, a positive relationship was revealed between these measures [24]. Critically, saliva concentrations reliably reflect oxytocin levels in the cerebrospinal fluid [25], which indicates that our measurement provided information about oxytocin activity in the central nervous system. However, plasma oxytocin measurements are not likely to be a feasible indicator of brain oxytocin activity [25].

The “social peptide” hypothesis supposes that oxytocin is specifically implicated in self-related and interpersonal processes, including mentalization, attachment, and affect regulation [10,12,26]. In accordance with this hypothesis, there was a link between interpersonal bonding and plasma oxytocin in depressed individuals [27], and saliva oxytocin levels predicted attentional orientation to social stimuli [28]. Intriguingly, fathers with disorganized attachment exhibited increased salivary oxytocin following the presentation of attachment-projecting pictures (free-response pictures designed to activate the human attachment system) [29].

How could results from studies using externally administered oxytocin confirm and extend the data obtained from baseline peripheral oxytocin measurements? Initial evidence suggested that externally administered oxytocin improved mentalization abilities when participants recognized social emotions by viewing the eye regions of faces [26]. Still, oxytocin turned out to improve mentalization only in individuals with low empathy scores [30], and oxytocin failed to improve mentalization abilities in women with schizophrenia [31].

Further clinical evidence indicates that the relationship between oxytocin and mentalization is multifaceted and depends on baseline mood: oxytocin administration led to opposite effects in reaction times on a mental state attribution task in depressed and control groups, with faster responses observed exclusively in the healthy control group [32]. Finally, a meta-analysis indicated that intranasal oxytocin administration enhanced recognition of basic emotions, particularly fear, and augmented positive but not negative emotion expression in non-clinical populations [33]. Unexpectedly, oxytocin had no significant effect on mentalization and did not seem clinically valuable for aiding mentalization in people with deficits in this skill, such as individuals with autism [34]. These results led researchers to question the utility of the intranasal oxytocin administration paradigm and required improving the accuracy and reliability of the oxytocin level evaluation following the administration [35,36].

However, beyond the “social peptide” hypothesis, it has been proposed that oxytocin is an allostatic and resilience hormone responsible for maintaining homeostatic and behavioral stability in challenging and stressful situations [37,38]. In this sense, the interaction between oxytocin and the hypothalamic–pituitary–adrenal (HPA) axis is essential, spotlighting cortisol secretion in the adrenal cortex in response to physiological cues and stress. The oxytocin system and the HPA axis appear to have a reciprocal influence on each other, partly determined by psychosocial factors [7,39,40]. Although oxytocin has anti-stress properties counteracting cortisol, higher levels of oxytocin are paradoxically detected in individuals with anxiety and interpersonal distress [12,41,42]. In a naturalistic stress situation (school performance), salivary oxytocin levels elevated several weeks before the semester’s end, followed by rising salivary cortisol levels. Higher baseline oxytocin levels were associated with positive feelings after the stress and better cognitive performance [43]. Morning salivary or plasma cortisol concentrations and affective control are associated in males, but empathy and emotion recognition abilities do not correspond with peripheral cortisol levels [44]. In fathers with disorganized attachment, higher salivary cortisol levels were found [29]. Altogether, while the HPA axis represents the biological basis of the “fight-or-flight” response, oxytocin (together with cannabinoids, opioids, and dopamine) might be a central factor in the “tend and befriend” response (affiliation under stress) [45,46].

The shortcomings of the literature outlined above indicate that it is indispensable to investigate the relationship between mentalization and endogenous oxytocin and cortisol levels in large and representative non-clinical populations, together with a detailed deconstruction of behavioral phenotypes. A possible tool for the mechanistic deconstruction of mentalization is the perception of biological motion (e.g., dynamic facial expression, body language expressing emotions, gait, posture, and walking) [47,48]. For example, developmental data from children suggest that the ability to perceive biological motion in noise correlates with mental state attribution based on eye regions of faces and also on the verbal interpretation of stories about different characters [48]. In addition, oxytocin enhances the perception of biological motion [49] and modulates brain rhythms during the processing of biological motion [50,51]. A single dose of intranasal oxytocin facilitates neuronal activity in the superior temporal sulcus implicated in the perception of biological motion [52]. According to this hierarchical model, biological motion perception is a low-level input that provides essential information to the mentalization system. First, the perceptual system detects biological motion. In the second stage, intentions are automatically attributed to others. Finally, the highest level of mentalization includes conscious self-reflection and awareness of mental states, as measured by the MZQ. Therefore, the efficacy of biological motion perception may predict higher-level mentalization (MZQ), and oxytocin may improve both biological motion perception and mentalization. Given the opposite role of oxytocin and cortisol, one could expect that cortisol disrupts biological motion perception and mentalization [7,39,40], although meta-analytic evidence failed to support a relationship between cortisol and mentalizing abilities [44].

Based on the literature highlighted above, we had the following main hypotheses:

1. In a representative group of non-clinical individuals from the general population, endogenous oxytocin levels predict mentalization abilities.
2. Better mentalization positively correlates with higher sensitivity to biological motion.
3. No correlation exists between cortisol levels, mentalization, and biological motion detection.
4. We also assessed the actual affective state of the participants. We hypothesized that positive emotions are associated with better mentalizing abilities.

## 2. Materials and Methods

### 2.1. Participants

We assessed 150 individuals from the general population (71 men, 79 women, all Caucasian). The average age was 39.4 years (*SD* = 13.7). The average number of years of education was 12.6 (*SD* = 7.8). The participants’ features are shown in Table 1. We used social media advertisement and a random digit dialing recruited survey to obtain a representative sample for age, gender, education, income, rural and urban geography, and perceived health (all Cramer V-values < 0.1) [53]. Individuals with psychiatric and neurological disorders were not included in the study. We assessed the participants individually in the laboratory. All questionnaires were administered in a pen-and-paper format. The order of task administration was counterbalanced across the participants.

Following a detailed description of the protocol, written informed consent was obtained. The study was approved by the National Medical Research Council (ETT-TUKEB 18814, Budapest, Hungary). We performed all procedures according to the relevant guidelines, regulations, and the Declaration of Helsinki.

### 2.2. Mentalization Questionnaire (MZQ)

The MZQ is a self-rated instrument to assess mental state attribution [54,55]. It comprises 15 items rated on a 1–5 scale (1: no agreement; 5: complete agreement). Sample items were the following: “Most of the time I don’t feel like talking about my thoughts and feelings with others; Sometimes I only become aware of my feelings in retrospect; Often I feel threatened by the idea that someone could criticize or offend me.” The total score ranges from 15 to 75, with higher scores indicating less efficient mentalization (deficient mentalizing score). In the original scale, the total score was divided into four subscales describing different aspects of mentalization (self-reflection, emotional awareness, psychic equivalence mode, and affect regulation). However, the factor structure is uncertain, so we use the total score in the statistical analysis (Cronbach’s alpha: 0.86) [55,56].

### 2.3. Biological Motion

**Figure 1:**
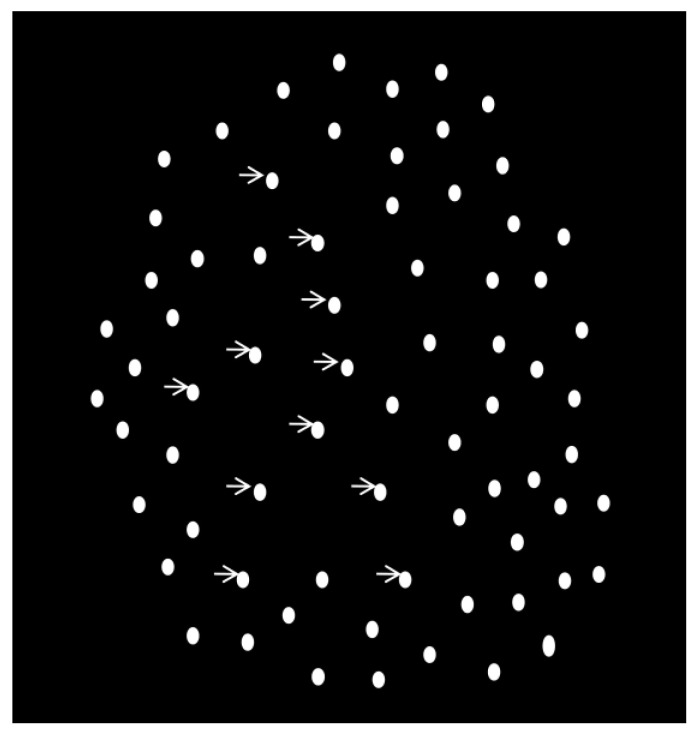
Illustration of the biological motion detection task. The white dots indicated by arrows comprise the walking character to be detected embedded in the noise dots.

As described previously, participants detected a walking human-like figure consisting of 11 white dots against a black background [49] (Figure 1). The 11 white signal dots were embedded in a cloud of 176 dynamic white mask dots. The task was to determine whether the walking figure appeared among mask dots or not by pressing two distinct keys (no: 0, yes: 1). A display trial depicted one step-cycle of walking (60 frames of motion, 42.5 frames/s) during which participants made the yes or no decisions. There were 100 trials, of which 50 contained the walking character and 50 trials consisted of noise. The dependent measure was sensitivity (*d’* = Z_hit rate_–Z_false alarm rate_) [49].

### 2.4. Positive and Negative Affect Schedule (PANAS)

Participants rated their present affective state for 20 emotions on a 1–5 scale (1: very slightly or not at all; 5: extremely) [57]. The PANAS separately delineated ten positive emotions (“attentive, active, alert, enthusiastic, excited, determined, inspired, interested, proud, and strong”) and ten negative emotions (“afraid, ashamed, distressed, guilty, hostile, irritable, jittery, nervous, scared, and upset”) [57]. We used the PANAS to characterize the participants’ actual affective state and contrast that with mentalization.

### 2.5. Saliva Cortisol and Oxytocin

Saliva samples were drawn between 4 p.m. and 6 p.m. using SalivaBio Passive Drool Method and stored at −20 °C. Free concentrations were measured using Salimetrics (assay range: 0.012–3.00 µg/dL; sensitivity: <0.007 µg/dL). We analyzed two samples with excellent consistency (<2% differences between the two samples).

We measured saliva oxytocin levels using the Oxytocin Enzyme Immunoassay kit according to the protocol of Enzo Life Sciences (Ann Arbor, MI, USA, cat. #900-153). We used Lambda Integrator to measure optical density at 405 nm (LAMBDA Instruments GmbH, Baar, Switzerland). The assay’s sensitivity is 11.6 pg/mL (range: 15–1000 pg/mL). The assay variations were acceptable (intra-assay: 3.2%, inter-assay: 5.3%).

The sample was screened for confounding factors (e.g., smoking, exercising before participation, hours of sleep in the previous night, being postmenopausal, and hormonal contraception), and these factors were included in the analysis as covariates according to current methodological recommendations [58].

### 2.6. Statistical Analysis

We used STATISTICA 13.3. (Tibco) and JASP 0.17.1. Following descriptive statistics (mean, standard deviation, and range), testing for normal distribution (Kolmogorov–Smirnov test), and homogeneity of variance (Levene’s test), Pearson’s product-moment correlation coefficients were calculated among the variables (MZQ deficient mentalization score, biological motion detection, PANAS scores, age, socioeconomic status, saliva cortisol, and oxytocin levels). We used multiple regression analysis to delineate the predictors of MZQ scores. Biological motion, PANAS, age, sex, socioeconomic status, saliva cortisol, and oxytocin were potential predictors, which were all included in the same regression model. Given that the relationship between oxytocin levels and behavioral measures was the cornerstone of our hypothesis, we conducted a median split analysis (low vs. high oxytocin levels) to test whether we could confirm the regression results with a categorical approach [59,60]. Two-tailed *t*-tests were used to compare MZQ deficient mentalization scores, biological motion detection, and cortisol levels in individuals with low vs. high oxytocin concentrations. The level of statistical significance was set at alpha < 0.05 (Bonferroni’s corrections for multiple comparisons in the case of correlation coefficients: *p* < 0.005). To determine statistical power, we used the squared multiple correlation and regression module of the STATISTICA 13.3 package. In addition to conventional statistics, we also used a Bayesian approach, with a particular reference to calculating the Bayes Factor (*BF*_10_ 1–3: weak evidence, 3–10: moderate evidence, >10: strong evidence). The Bayesian approach provides an alternative to conventional statistics by giving a joint probability distribution of the parameters (prior distributions combined with observational data) [61].

## 3. Results

### 3.1. Demographic Characteristics and Test Results

Table 1 depicts the means, standard deviations, and ranges of demographic measures, results from the questionnaires (MZQ and PANAS scores), biological motion sensitivity, and oxytocin and cortisol levels.

### 3.2. Correlation Coefficients

Table 2 depicts Pearson’s correlation coefficients. The statistical power was 0.95. Critically, following corrections for multiple comparisons, the MZQ deficient mentalizing scores correlated only with oxytocin levels (*r*(150) = −0.36, *p* < 0.001) and biological motion detection (*r*(150) = −0.32, *p* < 0.001. The *BF*_10_-values from the Bayesian correlation analysis are shown in Table 3.

### 3.3. Regression Analysis

Multiple regression analysis indicated that the MZQ deficient mentalizing scores were significantly predicted by oxytocin levels (β* = −0.24, *SE* = 0.15, *R*^2^ = 0.14, *p* = 0.003), PANAS-positive scores (β* = −0.22, *SE* = 0.13, *R*^2^ = 0.06, *p* = 0.004), and biological motion (β* = −0.24, *SE* = 0.59, *R*^2^ = 0.08, *p* = 0.002). In the inverse analysis, we tested whether oxytocin levels and biological motion detection also predict positive emotions. However, the PANAS-positive scores were predicted only by the MZQ scores (β* = −0.27, *SE* = 0.05, *R*^2^ = 0.20, *p* = 0.003) and not by oxytocin and biological motion (*p*s > 0.2). There were no significant predictors for PANAS-negative scores (*p*s > 0.1). Bayesian linear regression analysis confirmed that the oxytocin–PANAS-positive–biological motion interaction best predicted the MZQ deficient mentalizing scores (*P*(*M*) = 0.02, *BF*_10_ = 5.51). This interaction means that the highest variance of MZQ scores is explained when oxytocin levels, PANAS-positive scores, and biological motion detection are together included in the Bayesian regression model.

### 3.4. Median Split Analysis

We next performed a median split analysis using the oxytocin levels. Individuals with low vs. high oxytocin levels differed in cortisol levels (*M_low_* = 5.9, *SD* = 4.6; *M_high_* = 7.5, *SD* = 4.7; t (148) = −2.11, *p* = 0.04), MZQ deficient mentalizing scores (*M_low_* = 40.6, *SD* = 13.0; *M_high_* = 34.5, *SD* = 11.8; t (148) = 3.02, *p* = 0.003), and biological motion detection (*M_low_* = 3.29, *SD* = 1.54; *M_high_* = 3.97, *SD* = 1.67; t (148) = −2.60, *p* = 0.01).

**Figure 2:**
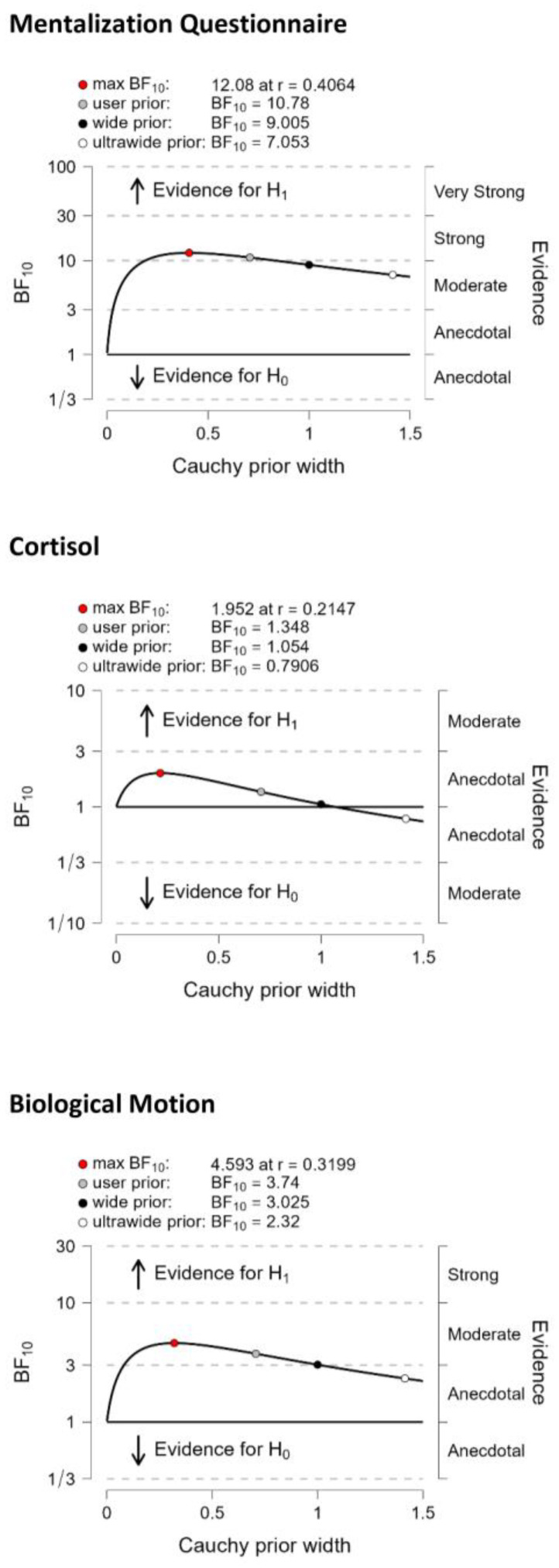
Bayes Factor (*BF*) robustness check from the median split analysis. H0—Hypothesis 0: no difference between the groups with high and low oxygen levels; H1—Hypothesis 1: the groups with high and low oxytocin levels significantly different.

Bayesian analysis revealed weak evidence that individuals with low oxytocin levels exhibited lower cortisol levels relative to participants with high oxytocin levels (*BF*_10_ = 1.4, error: 0.013%). However, there was moderate evidence for better biological motion detection in participants with high oxytocin levels than in those with low oxytocin levels (*BF*_10_ = 3.7, error: 0.006%) and strong evidence for better mentalization (lower MZQ scores) in people with high oxytocin levels than in those with low oxytocin levels (*BF*_10_ = 10.8, error: 0.003%) (Figure 2).

### 3.5. Comparison of Male and Female Participants

In addition to including sex in the regression analysis as a covariate, we also compared male and female participants with conventional and Bayesian *t*-tests. There were no significant differences between male and female participants in any measures included in this study (age, SES, MZQ, biological motion sensitivity, cortisol, and oxytocin) (*p*s > 0.2; *BF*_10_ < 1).

## 4. Discussion

The results of the present study indicate a positive relationship between saliva oxytocin, mentalization, and biological motion perception: individuals with higher endogenous oxytocin levels excelled on the mentalization self-report questionnaire (MZQ) and detected biological motion better when a walking dot-pattern character had to be noticed among noise dots. As expected, mentalization also predicted positive affectivity, but positive affectivity and oxytocin were independent, suggesting a specific link between oxytocin levels and mentalizing ability. In addition, we found that although mentalization predicted positive affectivity, oxytocin and biological motion failed to do so. In other words, oxytocin and biological motion are specific predictors of mentalization but not positive emotional experiences. It is important to note that only two correlations passed corrections for multiple comparisons: negative correlations between MZQ deficient mentalizing score and oxytocin and MZQ deficient mentalizing score and biological motion detection.

Although a meta-analysis has indicated that exogenous oxytocin does not affect mentalization skills [33], the relationship between endogenous oxytocin levels and mentalization still needs to be clarified. Surprisingly, the relationship between mentalization and oxytocin levels has not been assessed in a representative non-clinical sample to date, which our results show to be positively correlated. We included a large and representative non-clinical sample from the general population, filling an essential gap in the literature and providing supportive evidence for the association of endogenous oxytocin levels and mental state attribution abilities. This ability was independent of the subjective and transient experience of positive and negative emotions. However, results from a similarly large sample indicated that exogenously administered oxytocin did not affect mentalization performance and brain activation [62].

As outlined in the introduction, there is an inverse relationship between the physiological function of cortisol (pro-stress) and oxytocin (anti-stress). Paradoxically, we found a weak positive correlation between saliva cortisol and oxytocin. However, it is not rare in the literature. For example, Kuchenbecker et al. (2021) demonstrated a positive association between baseline cortisol and oxytocin levels before a cognitive challenge task [43]. Furthermore, in both experimental and naturalistic stress situations, both hormone levels increased, and oxytocin seemed to counteract and compensate for the effects of cortisol [43]. Importantly, we corrected our analysis for cortisol levels by including it in the regression model and demonstrated no significant relationships between cortisol, mentalization scores, and reported emotional experiences.

A particularly interesting issue is the role of biological motion perception in social cognition. The results of the present study show that a hierarchical structure can characterize the understanding of the mental state of others, from biological motion perception to the attribution of social emotions and intentions, because biological motion detection predicted the MZQ scores. Higher levels describe more complex and abstract functions, while lower levels explain how functions can be applied to specific stimuli and tasks [63]. Biological motion perception may be an entry-level building block of mental state attribution [47]. Rice et al. (2016) found that biological motion perception is significantly correlated with two measures of mentalization (mental state attributions based on facial eye regions and stories) in children aged 7 to 12 years [48]. This suggests that better performance in tasks measuring biological motion perception is associated with better performance in tasks measuring mentalization. Moreover, it should be noted that the study of Rice et al. (2016) did not find a correlation between biological motion perception and physical inferences from stories (e.g., “getting wet because a bush poked holes in his umbrella”), suggesting that biological motion perception is specifically linked to mental state inferences [48]. As children progress through the preschool age range, the accuracy of biological motion recognition continues to increase and is associated with social proficiency [64].

Grézes et al. (2001) demonstrated that biological motion could be perceived with just a few bright spots representing the body’s main joints in motion and that its perception activates the occipitotemporal junction and the left intraparietal cortex, respectively [65]. These findings suggest that specific brain regions may mediate biological motion perception and mentalization. In accordance with these findings, we observed a positive correlation between biological motion sensitivity and MZQ scores. Regarding the neurohormonal correlates, oxytocin levels were more closely associated with self-report mentalization scores than with biological motion detection, although externally administered oxytocin profoundly affects brain rhythms during biological motion perception [50,51]. The predictive effect of oxytocin on mentalization remained significant when biological motion perception was included in the analysis as a co-predictor.

Until recently, research on social cognition has paid scant attention to sex differences. It is now apparent, however, that social cognitive functions and their neuronal correlates markedly differ as a function of sex and gender [66,67,68]. Proverbio (2021) summarized that there are sex differences in “face processing, facial expression recognition, response to a baby schema, the ability to see faces in things, the processing of social interactions, the response to the other’s pain, interest in social information, processing of gestures and actions, biological motion, erotic, and affective stimuli. Sex differences in oxytocin-based parental response are also reported” [67]. Evidence suggests better mentalization in females than in males, which is led by results from mental state recognition studies based on eye regions of faces, although performance differences may be explained by gender as a social construct and not by biological sex [69]. This is consistent with results from investigations focusing on perspective taking, empathy, and emotional intelligence, indicating that these social cognitive measures are more closely associated with masculine and feminine gender roles than biological sex [70]. However, we found no significant differences between male and female participants on the MZQ, which is consistent with the findings of Riedl et al. (2023), who assessed the original and shortened scale versions [56]. It seems that the MZQ has low sensitivity to sex differences in mentalization. Finally, we did not record significantly higher oxytocin levels in females than in males, which contradicts a previous study using plasma samples [71]. The weak correspondence between saliva and plasma oxytocin levels may explain this discrepancy [25].

Despite the large and representative sample, this study is not without limitations, with a particular reference to the narrow scope of research. First, oxytocin and cortisol were measured only at a single time point, and we could not analyze the daily changes in their saliva concentrations. In addition, correlations do not allow one to infer a causal role. For example, it could be that oxytocin is higher in individuals who are also more adept at mentalizing because of greater vigilance, a calmer state of mind, or other trait and state markers without oxytocin itself being directly involved in the mentalizing process. Second, peripheral levels are only proxy measures of central nervous system activity, and complementary brain imaging measurements are warranted to confirm the indirect findings. Third, findings should be confirmed in an intervention study using externally administered hormones or modulating environmental circumstances (e.g., stress induction and relaxation). Fourth, mentalization is not a unitary construct; we need more tests to assess its facets. However, the measurement of mentalization subcomponents is challenging. Riedl et al. (2023) evaluated the psychometric properties of the MZQ in a large representative German population sample, including acceptance, reliability, and validity. The authors found that acceptance was good, but the internal consistencies and factor structure of the original four subscales were not acceptable, and the MZQ is a valid self-report instrument to delineate inner mental states. However, in non-clinical samples, the total score of the MZQ is recommended, and the subcomponents cannot be discriminated [56]. Finally, this study had a relatively limited scope with few variables. However, we intended to avoid type I errors and spurious correlations by limiting the number of variables and maintaining a hypothesis-driven approach.

## 5. Conclusions

In conclusion, our results show that peripheral oxytocin has been involved in social cognition. We demonstrated a positive relationship between mentalization abilities, biological motion perception, and peripheral oxytocin levels in humans from non-clinical samples. We also found a weak positive correlation between saliva cortisol levels and oxytocin. These results might motivate future studies to explore the causal relationship between mentalization, biological motion perception, and oxytocin (e.g., oxytocin may facilitate biological motion detection, which, in turn, may boost higher-level mental state attribution). The delineation of different levels of social information processing and its regulation by oxytocin might also facilitate clinical research to better understand the role of mentalization and oxytocin in neuropsychiatric disorders.

## Figures and Tables

**Table 1:** Characteristics of the participants and results from the assessments (*N* = 150).

	Mean	Standard Deviation	Range (Min–Max)
Age	39.4	13.7	18–77
Socioeconomic status (Hollingshead scale)	46.8	8.8	28–66
Mentalization Questionnaire deficient mentalizing score	37.7	17.8	15–75
PANAS-positive emotions score	19.9	7.6	10–47
PANAS-negative emotions score	21.4	8.0	10–44
Biological motion detection score (*d’*)	3.6	1.6	1.0–7.0
Saliva oxytocin level (pg/mL)	10.7	6.6	0–28
Saliva cortisol level (ng/mL)	6.6	4.7	0–24

PANAS—Positive and Negative Affective Schedule.

**Table 2:** Pearson’s product-moment correlation coefficients (*r*) between the variables included in the analysis.

	MZQ	BM	PANAS-p	PANAS-n	Age	SES	Oxytocin	Cortisol
**MZQ**	-	−0.32 **	−0.28 *	−0.09	0.12	0.08	−0.36 **	−0.18 *
**BM**	−0.32 **	-	0.10	0.00	−0.09	−0.20 *	0.20 *	0.05
**PANAS-p**	−0.28 *	0.10	-	0.16	−0.07	−0.08	0.15	−0.06
**PANAS-n**	−0.09	0.00	0.16	-	0.06	0.09	0.14	0.06
**Age**	0.12	−0.09	−0.07	0.06	-	0.01	−0.05	−0.13
**SES**	0.08	−0.20 *	−0.08	0.09	0.01	-	−0.03	0.04
**Oxytocin**	−0.36 **	0.20 *	0.15	0.14	−0.05	−0.03	-	0.27 *
**Cortisol**	−0.18 *	0.05	−0.06	0.06	−0.13	0.04	0.27 *	-

** *p* < 0.01 (Bonferroni-corrected threshold), * *p* < 0.05; MZQ—Mentalization Questionnaire scores, BM—biological motion detection scores’ (*d’*), PANAS-p—Positive and Negative Affective Schedule—positive emotion scores; n—negative emotion scores, SES—socioeconomic status (Hollingshead scale).

**Table 3:** Bayesian correlation results (*BF*_10_-values).

	MZQ	BM	PANAS-p	PANAS-n	Age	SES	Oxytocin	Cortisol
**MZQ**	-	252.2	32.5	0.2	0.3	0.2	2116.5	1.1
**BM**	252.2	-	0.2	0.1	0.2	1.8	1.9	0.1
**PANAS-p**	32.5	0.2	-	0.5	0.2	0.2	0.5	0.1
**PANAS-n**	0.2	0.1	0.5	-	0.1	0.2	0.4	0.1
**Age**	0.3	0.2	0.2	0.1	-	0.1	0.1	0.3
**SES**	0.2	1.8	0.2	0.2	0.1	-	0.1	0.1
**Oxytocin**	2116.5	1.9	0.5	0.4	0.1	0.1	-	31.5
**Cortisol**	1.1	0.1	0.1	0.1	0.3	0.1	31.5	-

## Data Availability

The raw data are available as Supplementary Material to this article.

## Supplementary Materials

The following supporting information can be downloaded at https://www.mdpi.com/article/10.3390/life13061329/s1, Raw Data.
